# Feasibility of an Inversion Recovery-Prepared Fat-Saturated Zero Echo Time Sequence for High Contrast Imaging of the Osteochondral Junction

**DOI:** 10.3389/fendo.2021.777080

**Published:** 2021-12-24

**Authors:** Hyungseok Jang, Yajun Ma, Michael Carl, Alecio F. Lombardi, Eric Y. Chang, Jiang Du

**Affiliations:** ^1^ Department of Radiology, University of California, San Diego, CA, United States; ^2^ GE Healthcare, San Diego, CA, United States; ^3^ Radiology Service, VA San Diego Healthcare System, San Diego, CA, United States

**Keywords:** UTE, ZTE, inversion recovery, osteoarthritis, cartilage, osteochondral junction

## Abstract

**Purpose:**

The osteochondral junction (OCJ) region—commonly defined to include the deep radial uncalcified cartilage, tidemark, calcified cartilage, and subchondral bone plate—functions to absorb mechanical stress and is commonly associated with the pathogenesis of osteoarthritis. However, magnetic resonance imaging of the OCJ region is difficult due to the tissues’ short transverse relaxation times (i.e., short T_2_ or T_2_*), which result in little or no signal with conventional MRI. The goal of this study is to develop a 3D adiabatic inversion recovery prepared fat saturated zero echo time (IR-FS-ZTE) sequence for high-contrast imaging of the OCJ.

**Method:**

An IR-FS-ZTE MR sequence was developed to image the OCJ on a clinical 3T MRI scanner. The IR-FS-ZTE sequence employed an adiabatic inversion pulse followed by a fat saturation pulse that suppressed signals from the articular cartilage and fat. At an inversion time (TI) that was matched to the nulling point of the articular cartilage, continuous ZTE imaging was performed with a smoothly rotating readout gradient, which enabled time-efficient encoding of the OCJ region’s short T_2_ signal with a minimal echo time (TE) of 12 μs. An ex vivo experiment with six cadaveric knee joints, and an *in vivo* experiment with six healthy volunteers and three patients with OA were performed to evaluate the feasibility of the proposed approach for high contrast imaging of the OCJ. Contrast-to-noise ratios (CNRs) between the OCJ and its neighboring femoral and tibial cartilage were measured.

**Results:**

In the *ex vivo* experiment, IR-FS-ZTE produced improved imaging of the OCJ region over the clinical sequences, and significantly improved the contrast compared to FS-ZTE without IR preparation (p = 0.0022 for tibial cartilage and p = 0.0019 for femoral cartilage with t-test). We also demonstrated the feasibility of high contrast imaging of the OCJ region *in vivo* using the proposed IR-FS-ZTE sequence, thereby providing more direct information on lesions in the OCJ. Clinical MRI did not detect signal from OCJ due to the long TE (>20 ms).

**Conclusion:**

IR-FS-ZTE allows direct imaging of the OCJ region of the human knee and may help in elucidating the role of the OCJ in cartilage degeneration.

## Introduction

Osteoarthritis (OA) is one of the most common diseases, afflicting 30 million people in the United States alone (1). The assessment of OA is largely focused on cartilage, a complexly structured tissue that is comprised of multiple layers—namely, the superficial, middle, and deep layers. The osteochondral junction (OCJ) region is commonly defined to include the interface between the subchondral bone and both the deep (radial) and calcified cartilage, and functions by absorbing mechanical stress between those regions. Recently, it has been reported that the OCJ is associated with the pathogenesis of OA, suggesting that assessment of the OCJ region may have potential as a new diagnostic tool for OA (2–4).

Magnetic resonance imaging (MRI) is a promising non-invasive imaging modality for the assessment of OA due to the excellent soft tissue contrast it offers. Conventional MRI sequences for imaging cartilage in the knee joint typically include fast spin echo (FSE) sequences with T_2_ or T_1_ weighting and with or without fat suppression techniques (5–8) such as short tau inversion recovery (STIR), spectral adiabatic inversion recovery (SPAIR), or chemical shift-based fat saturation. Recently, more advanced MR imaging techniques have been proposed to characterize knee cartilage, including balanced steady-state free precession (bSSFP) (9, 10), diffusion imaging (11, 12), and double echo steady-state (DESS) sequences (13, 14). Despite promising overall results in the knee images, the abovementioned MR techniques are not able to directly resolve tissues with short T_2_* relaxation times (< ~1 ms) in the OCJ region due to the sequences’ relatively long echo times (TEs) which are on the order of several milliseconds or longer.

Ultrashort echo time (UTE) imaging has been actively investigated as a promising approach for imaging those tissues with short T_2_* relaxation times. UTE imaging relies on the ability to shorten the TE by simply removing the rewinding gradient required in conventional Cartesian MR imaging, and to subsequently acquire center-out projection data. While the shortened TE could provide a number of imaging advantages, there are still several technical challenges in the UTE approach. First, TE is limited by the RF coil transmit/receive switching time, which is typically ~30-200 µs depending on the performance of both the MR system and the RF coil (15, 16). Second, the encoding efficiency and effective TE of UTE imaging is limited by the gradient slew rate, where an additional delay must be imposed to reach the maximum gradient amplitude that corresponds to the desired readout bandwidth (BW) (15–17). Zero echo time (ZTE) imaging is based on a different encoding strategy where a fully ramped-up, constant gradient with a short RF pulse enables more time-efficient encoding, which may be beneficial in imaging tissues with very short T_2_* relaxation times (18–20). However, some important imaging parameters such as the flip angle (FA) and the readout BW are often limited in ZTE imaging (21–24). Another limitation associated with UTE and ZTE imaging is the poor image contrast for tissues with short T_2_ relaxation times, largely due to the high signal from surrounding tissues with long T_2_ relaxation times

Recently, various magnetization preparation techniques such as inversion recovery (IR), magnetization transfer (MT), and T_1ρ_ have been explored in UTE imaging (25–33). Among them, adiabatic IR has shown promising results in achieving high contrast UTE imaging of tissues with short T_2_ relaxation times while achieving efficient suppression of tissues with long T_2_ relaxation times. This technique has been further utilized in many neuro and musculoskeletal imaging studies (26, 28, 32–34). More recently, it has been shown that adiabatic IR preparation followed by chemical shift-based fat saturation can provide simultaneous suppression of fat and cartilage tissues with long T_2_ relaxation times (35, 36). Given these recent results, a combination of adiabatic IR preparation and fat saturation with ZTE data acquisition could prove useful for high contrast imaging of the OCJ region.

In this study, we explored the feasibility and efficacy of ZTE imaging combined with adiabatic IR preparation and chemical shift-based fat saturation for volumetric imaging of the OCJ region. An ex vivo experiment with six cadaveric knee joints and an *in vivo* experiment with six healthy volunteers and three patients with OA were performed to evaluate the proposed adiabatic IR-prepared fat-saturated ZTE (IR-FS-ZTE) technique for imaging of the OCJ region in the human knee joint.

## Materials And Methods

### Inversion Recovery-Prepared Fat-Saturated Zero Echo Time Sequence

**Figure 1:**
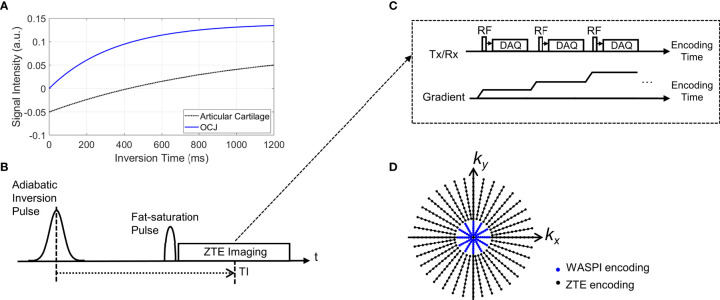
Pulse sequence diagram for IR-FS-ZTE. **(A)** An example of typical inversion recovery with an adiabatic inversion pulse, **(B)** signal preparation, **(C)** ZTE imaging, and **(D)** a 2D example of the k-space trajectory (black dots: high-resolution ZTE encoding, blue dots: low-resolution WASPI encoding). As shown in **(B)**, the adiabatic inversion pulse is followed by a fat saturation pulse that simultaneously suppresses the water signal (which has a long T_2_ relaxation time) and fat signal, which improves contrast and dynamic range of the targeted OCJ region.

**Figure 1A** illustrates typical signal inversion recovery curves for articular cartilage and the OCJ region. Due to its short T_2_* relaxation time (< ~1 ms), the longitudinal magnetization of the OCJ is not inverted by the relatively long adiabatic IR pulse (pulse duration ~10 ms), but partially inverted or saturated. Meanwhile, articular cartilage has a T_2_* relaxation time that is much longer than the duration of the adiabatic IR pulse, so its longitudinal magnetization is fully inverted. By selecting an inversion time (TI) that is tuned to the nulling point of articular cartilage, the OCJ region can be selectively imaged with excellent contrast and dynamic range. **Figure 1B** shows a pulse sequence for the signal preparation, where an adiabatic IR pulse is followed by chemical shift-based fat saturation to suppress signals from the articular cartilage and marrow fat simultaneously. ZTE imaging is then performed immediately after the fat saturation pulse (**Figure 1C**). Note that ZTE imaging benefits from short RF excitation to shorten a minimum TE, and fully ramped-up readout gradients to shorten an effective TE targeting rapidly decaying signal from short T_2_ components (20–22, 37). To speed up the data acquisition, multiple spokes are continuously acquired with a smoothly ramping (or rotating) readout gradient, as shown in **Figure 1C**. Unfortunately, ZTE encoding inevitably leaves a hole of missing data in the encoded central k-space due to the RF coil deadtime (a blind time during RF transmit/receive mode switching). In this study, the hole was filled with additional encoding with a reduced readout gradient amplitude, similar to the strategy used in Water- And Fat-Suppressed Proton Projection MRI (WASPI) (38, 39), as indicated with blue dots in **Figure 1D**. A WASPI factor, defined as the ratio between the gradient amplitude of ZTE and WASPI encoding, was introduced to control the size of the central k-space data to be acquired in the second acquisition.

### MR Imaging

The 3D IR-FS-ZTE sequence was implemented on a 3T clinical MR system (MR750, GE Healthcare, Milwaukee, WI, US). To evaluate the proposed method, six cadaveric human knee joints were scanned ex vivo. Additionally, six healthy volunteers (males, aged 35.3 ± 1.9) and three OA patients (males, aged 52.0 ± 3.7) were scanned in compliance with the Human Research Protection Program (HRPP) of the University of California, San Diego. All MR imaging was performed using an 8-channel transmit/receive knee coil (GE Healthcare).

*Ex vivo* imaging was performed using IR-FS-ZTE with the following parameters: an adiabatic Silver-Hoult inversion pulse (duration = 8.64 ms, BW = 1.5 kHz), a GE standard fat saturation pulse (duration = 16 ms, offset frequency = -440 Hz, BW = 500 Hz), TR = 1200 ms, TE = 12 μs, TI = 520 ms, FA = 8 °, readout BW = 62.5 kHz, field-of-view (FOV) = 130x130x80 mm^3^, acquisition matrix = 256x256x40, slice thickness = 2 mm, RF-to-RF timing (tau) = 2.3 ms, total number of spokes (TNSP) = 30338, number of spokes per IR (NSP) = 24, WASPI factor = 8, and scan time = 25 min 20 sec. For the first knee sample, an expanded IR-FS-ZTE imaging was performed with TIs = 200, 300, 420, 520, and 700 ms. ZTE with fat saturation but without IR preparation (FS-ZTE) was also performed for comparison using parameters matched with those of IR-FS-ZTE except for a reduced scan time of 1 min 41 sec.

*In vivo* imaging was performed using the following sequences: 1) IR-FS-ZTE with the same parameters as ex vivo imaging except matrix = 220x220x40, TI = 600 ms, tau = 1.9 ms, TNSP = 17940, NSP = 36, and scan time = 9 min 58 sec; 2) T_1_-weighted fast spin echo (T_1_w-FSE): FA = 140 °, TR = 4818 ms, TE = 28.4 ms, FOV = 130×130 mm^2^, matrix = 352×256, slice thickness = 2 mm, number of slices = 40, acceleration factor = 2, and scan time = 2 min 30 sec; 3) T_2_-weighted fast spin echo (T_2_w-FSE): GE standard fat saturation, FA = 140 °, TR = 9461 ms, TE = 72.5 ms, FOV=130×130 mm^2^, matrix = 352×256, slice thickness = 2 mm, number of slices = 40, acceleration factor = 2, and scan time = 2 min 32 sec.

### Data Processing

IR-FS-ZTE and FS-ZTE images were reconstructed using online reconstruction based on GE Orchestra SDK v1.7.1. In ZTE, the low-resolution k-space data acquired using WASPI were combined with high-resolution data using a linear merging filter with a transition duration of two data points. The density function was analytically calculated based on the inter-spoke distance and intra-spoke sampling density. For gridding, the following parameters were used: alpha = 2 and kernel width = 3 data points. The reconstructed images in each RF receiver channel were combined using the weighted sum of squares method in which the weighting factors were calculated based on the noise power in each channel.

For all *ex vivo* subjects, contrast-to-noise ratios (CNRs) between the OCJ and its neighboring femoral and tibial cartilage were measured in FS-ZTE and IR-FS-ZTE images. CNR was calculated by taking the absolute difference of average signal intensities in two regions normalized by standard deviation of background noise. Each ROI was manually segmented by a researcher with 10 years of research experience in musculoskeletal MRI under the supervision of a radiologist with over 20 years of experience. A student’s t-test was performed between the CNRs measured in the FS-ZTE and IR-FS-ZTE images, with a p-value of 0.05 considered statistically significant. For *in vivo* subjects, CNRs were only measured for IR-FS-ZTE because FS-ZTE images were not acquired. CNR measurement was not performed in the clinical MR images because no direct signal from OCJ was captured.

MRI osteoarthritis knee score (MOAKS) was used to semi-quantitatively score cartilage degeneration for all ex vivo subjects and *in vivo* OA patients (40). The knee was divided into fourteen subregions (medial e lateral patella; medial and lateral trochlea, central, and posterior femur; medial and lateral anterior, central, and posterior tibial plateau) for scoring of articular cartilage. The articular cartilage was assessed on the sagittal T_2_w-FSE MRI for both lesion size (any cartilage loss) and degree of full-thickness loss (if present): grade 0 (none); grade 1 (<10%); grade 2 (10-75%); grade 3 (>75%).

## Results

### *Ex Vivo* Study

**Figure 2:**
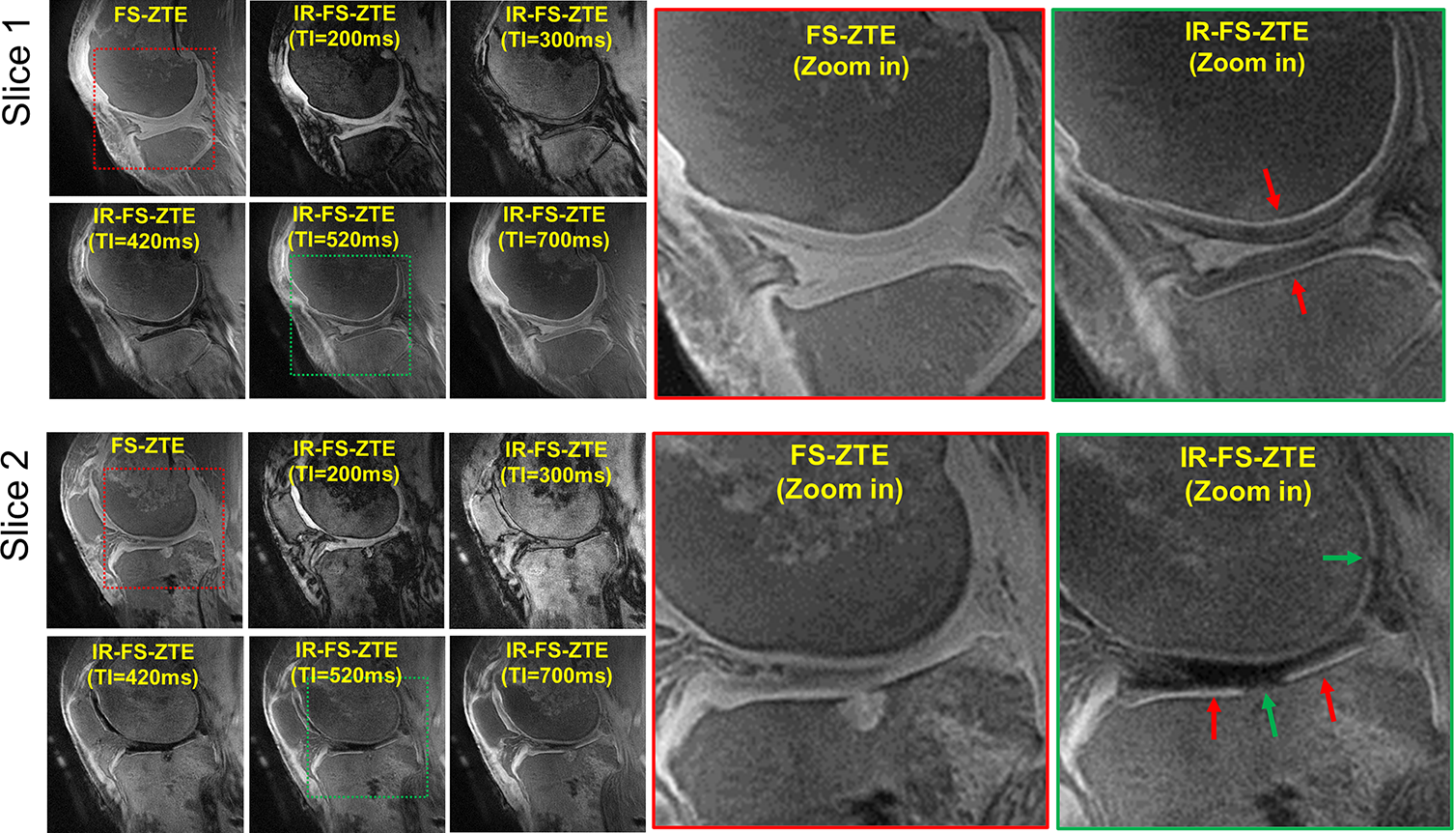
*Ex vivo* experiment with a knee joint sample (from a 71-year-old male donor). Two representative slices are shown to demonstrate the efficacy of inversion recovery preparation in OCJ imaging. IR-FS-ZTE with a TI of 520 ms shows the best image contrast, where the OCJ is well-delineated and represented by a bright line (red arrows), which is not obvious in FS-ZTE without inversion recovery preparation. Complete-thickness cartilage erosions involving the OCJ in the tibial plateau and posterior femoral condyle are better seen on the IR-FS-ZTE sequence compared to the FS-ZTE sequence, visualized as interruption of the bright line (green arrows).

**Figure 3:**
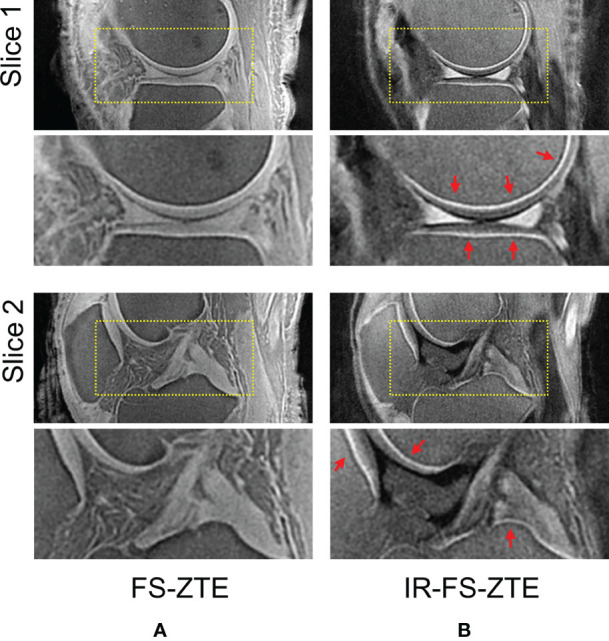
*Ex vivo* experiment with a knee joint sample (from a 48-year-old male donor). Two representative slices with **(A)** FS-ZTE and **(B)** IR-FS-ZTE. IR-FS-ZTE shows improved OCJ contrast compared to FS-ZTE, as indicated by red arrows and represented by the bright line.

**Figure 2** shows IR-FS-ZTE images from a representative ex vivo knee sample (from a 71-year-old male donor) acquired with five different TIs (200, 300, 420, 520, and 700 ms) compared with FS-ZTE images without adiabatic IR preparation. IR-FS-ZTE images with TI = 520 ms showed the best contrast for the OCJ region, suppressing signals from both articular cartilage and bone marrow while preserving signals from the OCJ region. Compared to FS-ZTE, IR-FS-ZTE provided improved morphology of not only the OCJ region (red arrows) but also of regional loss in the OCJ (green arrows). **Figure 3** shows a comparison between FS-ZTE and IR-FS-ZTE from another ex vivo knee sample (from a 48-year-old male donor). IR-FS-ZTE showed good contrast for the OCJ region and other tissues with short T_2_* relaxation times, such as the menisci in the knee joint, compared to FS-ZTE without IR preparation, as indicated by red arrows.

For all *ex vivo* knee samples, IR-FS-ZTE showed high contrast for the OCJ. In the FS-ZTE images, the CNRs measured between the OCJ and the femoral and tibial cartilage were 4.9 ± 3.1 and 9.0 ± 3.7, respectively. In the IR-FS-ZTE images, CNRs measured between the OCJ and the femoral and tibial cartilage were 13.5 ± 1.6 and 18.4 ± 6.0, respectively. In the t-test, IR-FS-ZTE showed significantly improved CNRs in both tibial (p = 0.0022) and femoral cartilage (p = 0.0019) compared to FS-ZTE without IR preparation.

Among the six knee samples, knee sample 1 (71-year-old male donor, shown in **Figure 2**) had a particularly high degree of cartilage degeneration, presenting with a total of eight subregions with full-thickness cartilage loss across at least 50% of the surface. Their combined MOAK scores were 62, 13, 20, 9, 2, and 3, respectively (**Table 1**).

**Table 1:** Combined cartilage MOAKS for each patient and *ex vivo* knee sample.

Subject	Combined Cartilage Score
Patient 1 (47M)	11
Patient 2 (56M)	36
Patient 3 (53M)	15
Knee Sample 1 (71M)	62
Knee Sample 2 (48M)	13
Knee Sample 3 (64M)	20
Knee Sample 4 (57M)	9
Knee Sample 5 (72M)	2
Knee Sample 6 (25M)	3

### *In Vivo* Study - Healthy Volunteers

For all healthy volunteers, IR-FS-ZTE yielded high contrast images of the OCJ, where morphology was well-delineated. CNRs measured between the OCJ and the femoral and tibial cartilage were 16.8 ± 3.9 and 16.8 ± 7.5, respectively.

**Figure 4:**
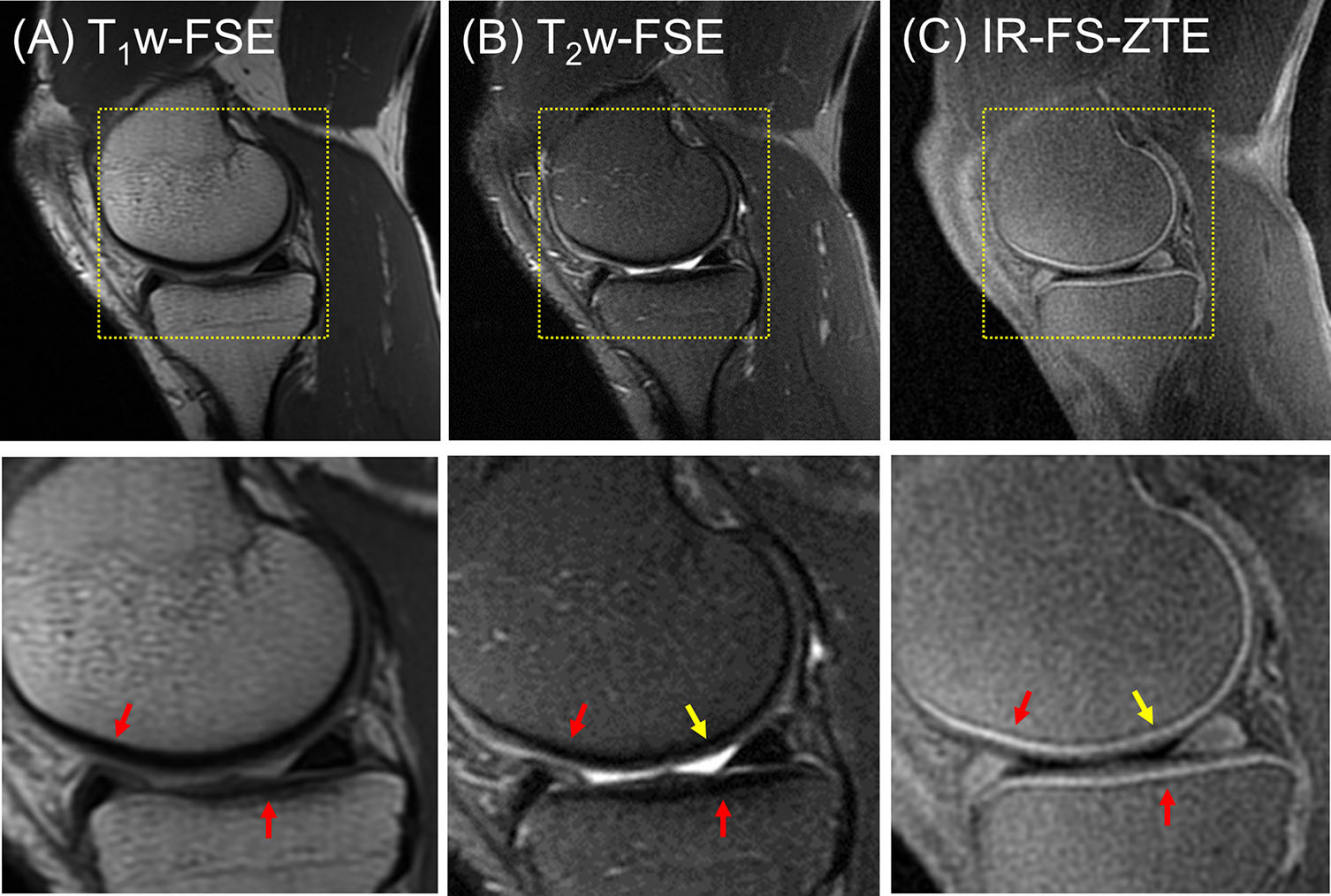
A healthy volunteer (35-year-old male). **(A)** T_1_w-FSE, **(B)** T_2_w-FSE, and **(C)** IR-FS-ZTE images (top) and the corresponding zoomed-in images (bottom). The short T_2_ signal from the OCJ region is resolved with high contrast in IR-FS-ZTE imaging **(C)**, while the signal is not captured at all by the conventional clinical MR imaging sequences **(A, B)**, as indicated by red arrows. The joint fluid with long T_1_ and T_2_ appears bright in T_2_-FSE imaging, but dark in IR-FS-ZTE imaging (yellow arrow).

**Figure 4** shows IR-FS-ZTE (**Figure 4C**), clinical T_1_w-FSE (**Figure 4A**), and T_2_w-FSE (**Figure 4B**) images from a representative healthy volunteer (35-year-old male). While the OCJ region was invisible in T_1_w-FSE and T_2_w-FSE images as a result of the sequences’ long TEs and the fast T_2_* decay for the OCJ, the proposed IR-FS-ZTE sequence showed high signal intensity and high contrast for the OCJ region, as indicated by red arrows. T_2_w-FSE showed bright contrast for fluid with long T_2_ relaxation times, whereas IR-FS-ZTE showed dark contrast for the same fluid, as indicated by yellow arrows.

### *In Vivo* Study - OA Patients

For all three OA patients, IR-FS-ZTE showed high contrast for the OCJ region. CNRs measured between the OCJ and the femoral and tibial cartilage were 15.4 ± 3.9 and 16.3 ± 2.5, respectively. The combined subregional cartilage MOAK scores for the three patients were 11, 36, and 15, respectively (**Table 1**).

**Figure 5:**
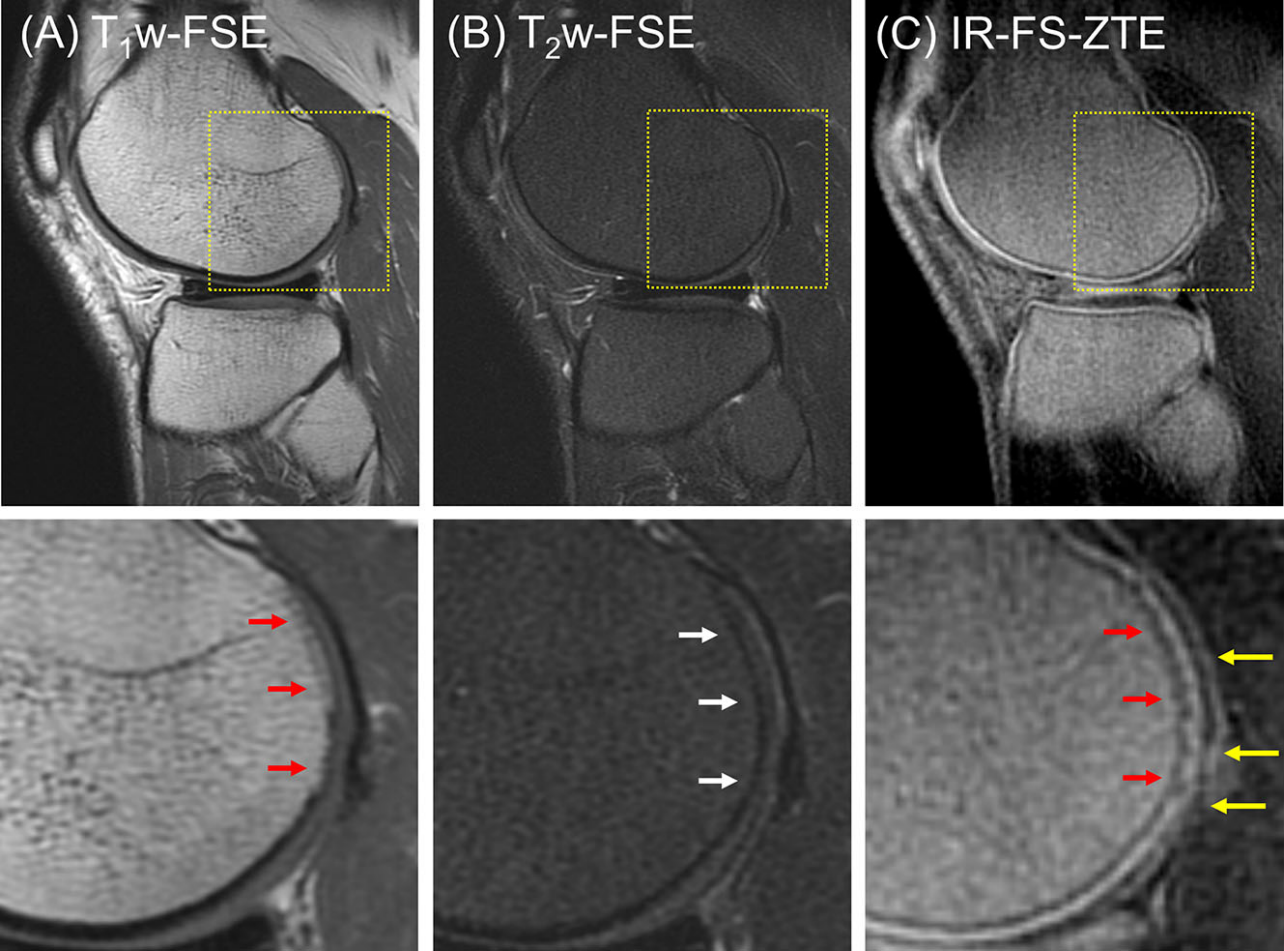
A patient with OA (47-year-old male). **(A)** T_1_w-FSE, **(B)** T_2_w-FSE, and **(C)** IR-FS-ZTE images (top) and the corresponding zoomed-in images (bottom). The T_1_w-FSE sequence shows only subtle subchondral bone irregularities in the posterior region of the femoral condyle (red arrows). The T_2_w-FSE sequence cannot detect subchondral or cartilage abnormalities (white arrows). The IR-FS-ZTE sequence, however, can show both subchondral bone and cartilage abnormalities (red and yellow arrows).

In the clinical reading, the first patient (a 47-year-old male) did not present any regions of full-thickness cartilage loss, although a combination of lesser degree cartilage degeneration throughout different subregions was observed (combined MOAKS = 11). **Figure 5** shows the MR images from the patient. The T_1_w-FSE sequence showed only subtle subchondral bone irregularities in the posterior region of the femoral condyle (red arrows). The T_2_w-FSE sequence was unable to detect subchondral or cartilage abnormalities (white arrows). The IR-FS-ZTE sequence, however, was able to show abnormalities in both the subchondral bone and cartilage (red and yellow arrows). Note the varying thickness of cartilage in the posterior femoral condyle (yellow arrows), the subchondral bone protrusions into the deep cartilage (red arrows), and the irregularities in the interface between the deep and superficial cartilage.

**Figure 6:**
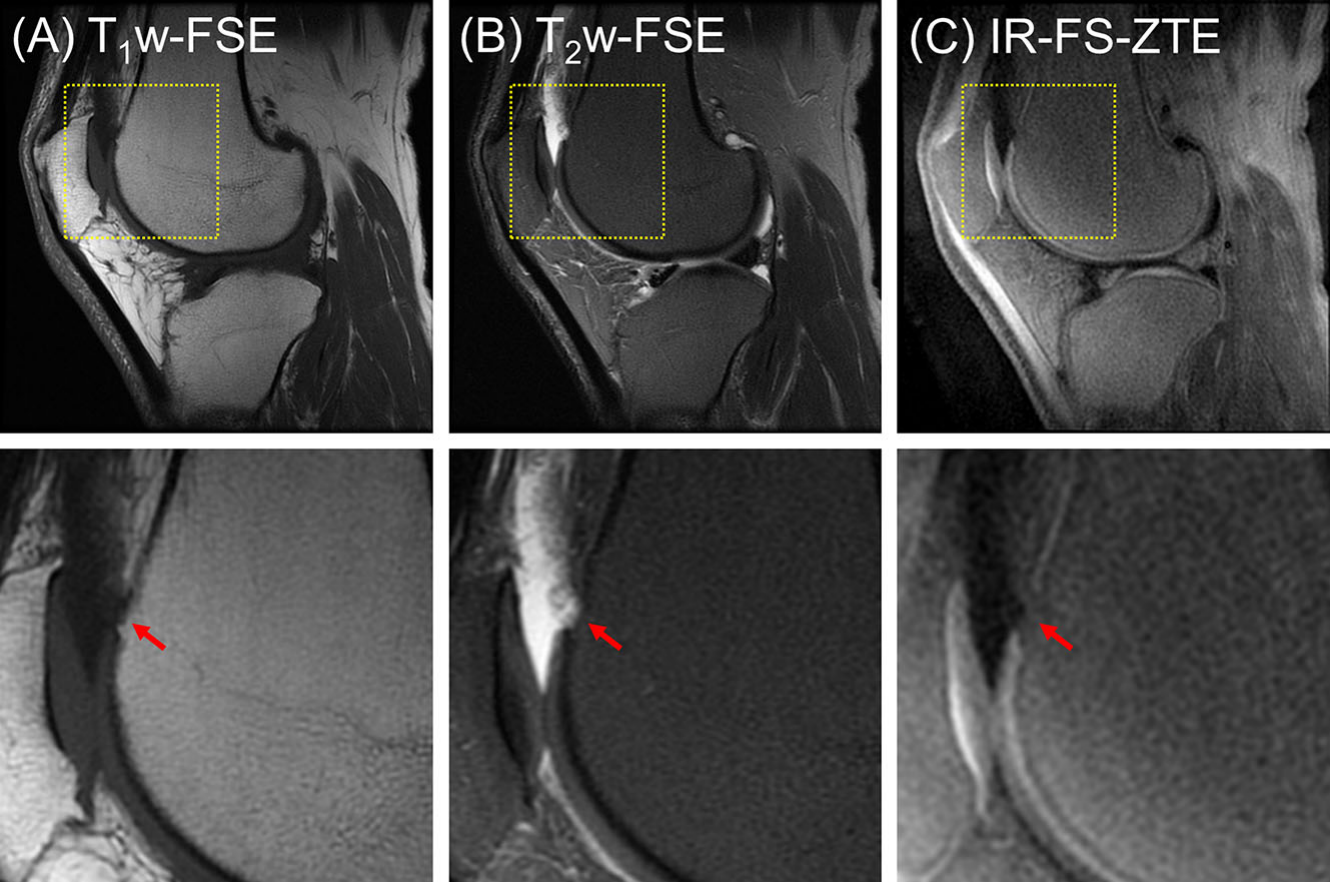
A patient with OA (56-year-old male). **(A)** T_1_w-FSE, **(B)** T_2_w-FSE, and **(C)** IR-FS-ZTE images (top) and their corresponding zoomed-in images (bottom). Regional loss of OCJ is well-delineated with IR-FS-ZTE **(C)**, whereas the lesion is obscured in clinical images **(A, B)**, as indicated by red arrows.

**Figure 7:**
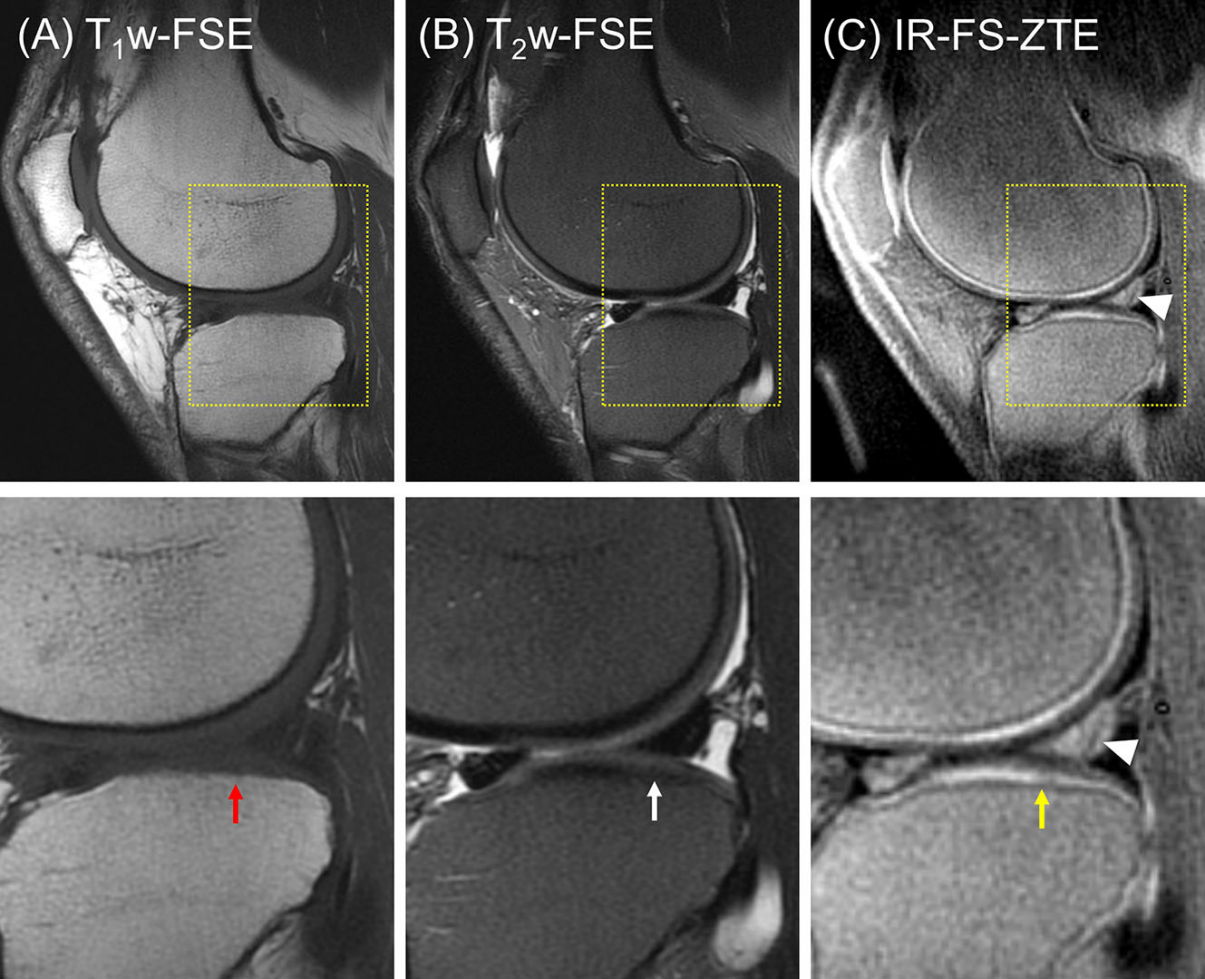
A patient with OA (56-year-old male). **(A)** T_1_w-FSE, **(B)** T_2_w-FSE, and **(C)** IR-FS-ZTE images (top) and the corresponding zoomed-in images (bottom). The T_1_w- and T_2_w-FSE sequences do not show any evident abnormality in the interface between the cartilage and subchondral bone in the lateral tibial plateau (red and white arrows), whereas the IR-FS-ZTE sequence highlights the OCJ, allowing for the visualization of a small signal abnormality in the deep cartilage (yellow arrow) as well as the altered signal in the posterior horn of the lateral meniscus (white arrowheads).

The second patient (a 56-year-old male) presented with three subregions of full-thickness cartilage loss greater than 75% of the articular surface and one subregion with a full-thickness loss involving 10-75% of the articular surface, reflecting a high degree of cartilage degeneration (combined MOAKS = 36). **Figure 6** shows the MR images from the patient. Regional loss of articular cartilage was well-delineated in both T_1_w-FSE and T_2_w-FSE images, as indicated by red arrows in **Figures 6A, B**, but it was not enough to directly reveal lesions in the OCJ region due to the poor contrast with which both normal and abnormal OCJ appeared. On the other hand, IR-FS-ZTE directly detected degeneration in the OCJ region as a discontinued line, as shown in **Figure 6C** (red arrow). **Figure 7** shows images with T_1_- and T_2_-weighting and IR-FS-ZTE from the same patient. The T_1_w- and T_2_w-FSE sequences (**Figures 7A, B**) did not show any evident abnormalities in the interface between the cartilage and subchondral bone in the lateral tibial plateau (red and white arrows). On the IR-FS-ZTE sequence (**Figure 7C**), however, the highlighted OCJ allowed for the visualization of a small signal abnormality in the deep cartilage (yellow arrow) that may have represented a cartilage calcification or protrusion from the subchondral bone. There was also a notably altered signal in the posterior horn of the lateral meniscus (white arrowheads), representing degeneration and which was better visualized on the IR-FS-ZTE sequence.

**Figure 8:**
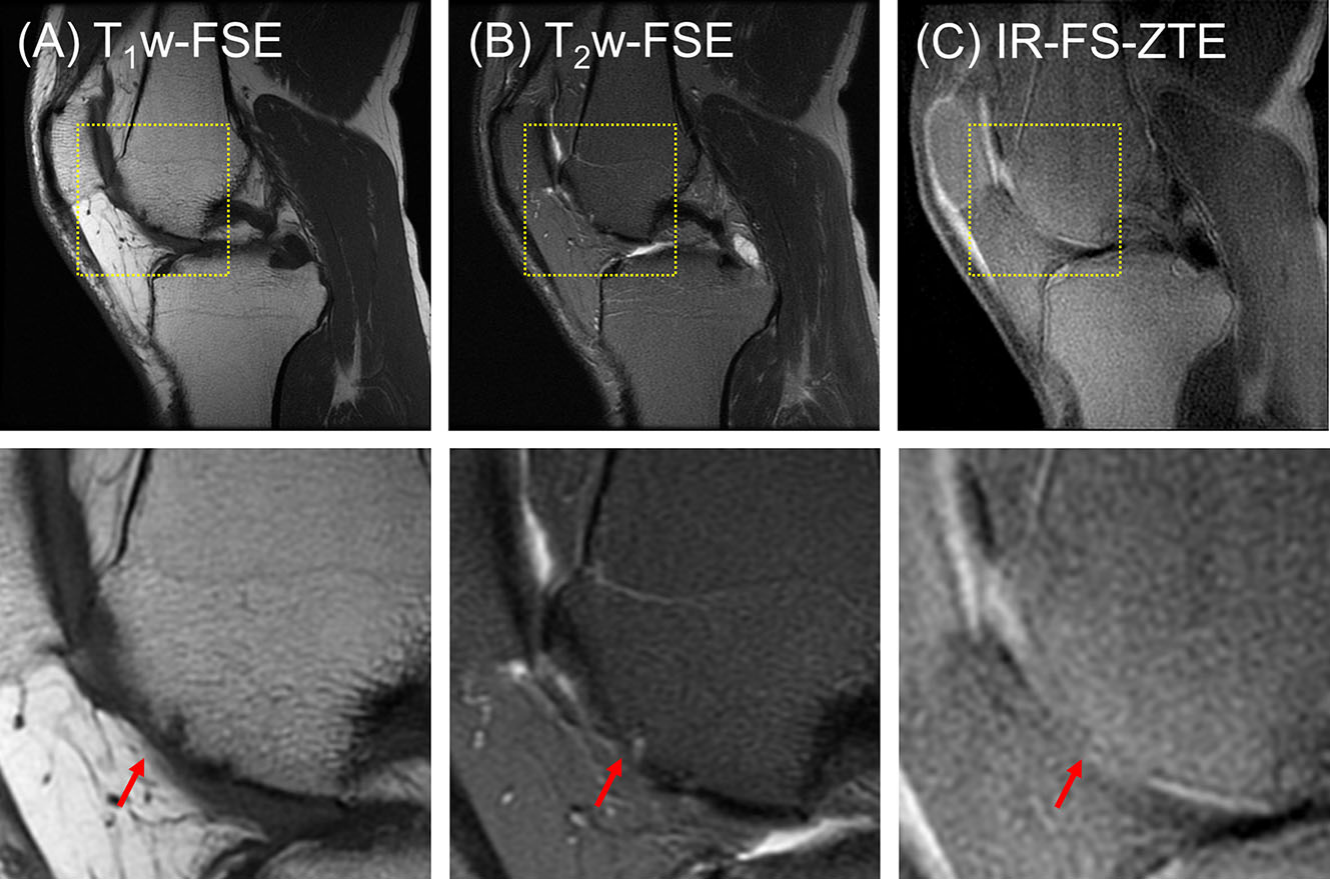
A patient with OA (53-year-old male). **(A)** T_1_w-FSE, **(B)** T_2_w-FSE, and **(C)** IR-FS-ZTE images (top) and the corresponding zoomed-in images (bottom). Focal complete-thickness cartilage erosion involving the OCJ and represented by interruption of the bright line in the femoral trochlea is detected in the IR-FS-ZTE image **(C)**, whereas the clinical images provide only indirect information of the lesion due to poor contrast for the OCJ region **(A, B)**, as indicated by red arrows.

The third patient (a 53-year-old male) presented with one subregion of full-thickness cartilage loss involving 10-75% of the articular surface (combined MOAKS = 15). **Figure 8** shows the images from the patient. Focal complete-thickness cartilage erosion involving the OCJ (represented by interruption of the bright line in the femoral trochlea) was well-depicted with the proposed IR-FS-ZTE sequence, as indicated by a red arrow in **Figure 8C**, but invisible in the clinical T_1_w-FSE and T_2_w-FSE images, as indicated by red arrows in **Figures 8A, B**. This was because the image contrast was not specific to the OCJ region.

## Discussion

In the literature, it has been reported that degeneration of the OCJ is commonly associated with the pathogenesis of OA. In OA, osteoclasts are activated in cartilage and subsequently form channels to the subchondral bone plate, triggering angiogenesis and peripheral nerve innervation from the bone marrow to the deep cartilage (2, 3). This is associated with a cascade of abnormalities including local inflammation and upregulation of metalloproteinase activity, degradation of the extracellular matrix, impairment of cartilage load-bearing capacity, and other degenerative changes (3, 4, 41). Characterization of the OCJ region is therefore of high interest and importance in the assessment of OA. We showed that the proposed IR-FS-ZTE sequence can directly capture signal from the OCJ region with significantly improved image contrast and dynamic range as a result of the proposed technique’s adiabatic IR preparation and fat suppression. This directly obtained morphological information may substantially improve the clinical diagnosis of lesions in the OCJ region.

The signal source in IR-FS-ZTE imaging of the OCJ remains to be investigated. It is likely that the deepest radial layer of articular cartilage, the calcified cartilage, and the subchondral bone plate all contribute to the IR-FS-ZTE signal. The calcified cartilage has a short T_2_* of around 2.0 ms (42), and the subchondral bone plate is believed to have a T_2_* close to that of cortical bone, which has been reported to have an extremely short T_2_* of around 1.0 ms or less (43). Therefore, the IR-FS-ZTE sequence’s TE of 12 µs should be able to directly detect signals from these tissue regions. However, a recent study by Nykanen et al. found that the bright signal line seen in UTE sweep imaging with Fourier transformation (SWIFT) imaging of cartilage samples resided within the deep radial noncalcified cartilage (44). This is likely due to the low proton densities of the calcified cartilage and subchondral bone, as well as their extremely short T_2_*s, which together result in a very low signal intensity in SWIFT imaging. Considering that the calcified cartilage and subchondral bone have short T_2_*s as well as short T_1_s (35), higher T_1_ weighting with a shorter TR and a higher flip angle may further increase their signal contribution. Higher RF power and stronger gradient strength are also helpful for direct imaging of the OCJ region. Clearly more research is needed to systematically investigate the effects of RF power, gradient strength, spatial resolution, as well as T_1_, T_2_*, and PD weighting on IR-FS-ZTE imaging of the OCJ region. Validation by histology and μCT would provide strong corroboration of the signal sources.

A potential downside of the IR-FS-ZTE technique is that the range of available FAs is limited due to the prescribed readout BW and to the excitation-readout scheme where a readout gradient is turned on before RF excitation. This approach is beneficial in imaging tissues with short T_2_ relaxation times because an effective TE that is near to zero can be achieved. However, this has the potential to limit the attainable signal-to-noise ratio. However, in this study we showed that IR-FS-ZTE can achieve high-quality images of the OCJ region, owing to the short tau (2.3 ms in the ex vivo protocol and 1.9 ms in the *in vivo* protocol) and high NSP (24 in the ex vivo protocol and 36 in the *in vivo* protocol) which allow oversampling (2x in the ex vivo protocol and 1.6X in the *in vivo*) to enhance the signal-to-noise ratio. Another limitation is that slab selection is not compatible with ZTE, which may limit the utilization of IR-FS-ZTE in body imaging where slab selection is desired. In applications where slab selection is required or desired such as spine imaging, spatial saturation technique can be utilized to improve image quality in IR-FS-ZTE imaging of the cartilaginous endplate (CEP).

More advanced techniques may be applied to the current IR-FS-ZTE sequence to further improve image quality for the OCJ. Interleaved encoding has recently been proposed to improve image quality and scan efficiency in IR-based hybrid UTE encoding (i.e., a mixture of Cartesian single point imaging (SPI) and radial frequency encoding), where SPI encoding is interleaved near the best nulling point (31). This approach significantly reduced imaging artifacts and improved image contrast in IR-based UTE imaging by assuring optimal nulling of targeted tissues in the center of k-space, which is the major contributor to image contrast. IR-FS-ZTE can also benefit from this strategy by interleaving WASPI encoding near the nulling point of articular cartilage. Another potential technique that may improve IR-FS-ZTE is frequency sweeping (or phase-modulated) RF excitation. Schieban et al. have recently shown the feasibility and efficacy of a short hyperbolic secant (HSn) pulse to achieve improved FA with reduced blurriness in ZTE imaging (23), which may also be an effective approach in IR-FS-ZTE-based OCJ imaging. We will further investigate this possibility in future studies.

This study has several limitations. First, only focused sets of imaging parameters were investigated in the MR experiments. There are more parameters that directly contribute to image quality in IR-FS-ZTE such as NSP, FA, and view ordering (31). Further investigation will be performed on those parameters in our future studies. Second, the current implementation of IR-FS-ZTE is based on a single IR (SIR) technique. As T_1_ may vary in articular cartilage, SIR may not be able to evenly suppress the tissues with long T_2_ relaxation times. The combination of a shorter TR and TI or the use of a dual IR technique could be used to address this challenge (27, 45), which will be further investigated. Third, no systematic comparison between IR-FS-ZTE and IR-FS-UTE was performed. Since UTE and ZTE are based on different acquisition and image reconstruction schemes, it is not trivial to perform fair comparison. It is still unknown whether the bright signal line represents the deep radial layer of cartilage, the calcified cartilage, the subchondral bone, or their combination. A systematic comparison requires further optimization of each sequence based on the T_1_, T_2_* and proton density of the OCJ (and its tissue components), which are largely unknown. Lastly, only a limited number of healthy volunteers and OA patients were scanned for our *in vivo* experiment. The efficacy of IR-FS-ZTE imaging of the OCJ region in the clinical assessment of OA must be further investigated through the recruitment of more patients who have differing degrees of degeneration.

In this study, we implemented the IR-FS-ZTE sequence on a clinical 3T MR system and showed its feasibility in high contrast OCJ imaging. IR-FS-ZTE showed improved image contrast for the OCJ region compared to clinical and FS-ZTE sequences. This technique can detect morphological changes in the OCJ region and its involvement in cartilage degeneration.

## Data Availability Statement

The raw data supporting the conclusions of this article will be made available by the authors, without undue reservation.

## Ethics Statement

The studies involving human participants were reviewed and approved by UCSD Human Research Protections Program. The patients/participants provided their written informed consent to participate in this study.

## Author Contributions

Implementation of MRI sequences: HJ, YM, and MC. Study Design: HJ, YM, and JD. Data collection: HJ, YM, and AL. Data interpretation: HJ, AL, EC, and JD. Manuscript writing: HJ, AL, EC, and JD. All authors contributed to the article and approved the submitted version.

## Funding

The authors acknowledge grant support from the NIH (R01 AR075825, R01 AR062581, R01 AR068987, R01 AR078877, R21 AR075851, and P30 AR073761), VA Clinical Science Research and Development (Merit Awards I01CX001388 and I01RX002604), and GE Healthcare.

## Conflict of Interest

MC was an employee of GE Healthcare.

The remaining authors declare that the research was conducted in the absence of any commercial or financial relationships that could be construed as a potential conflict of interest.

## Publisher’s Note

All claims expressed in this article are solely those of the authors and do not necessarily represent those of their affiliated organizations, or those of the publisher, the editors and the reviewers. Any product that may be evaluated in this article, or claim that may be made by its manufacturer, is not guaranteed or endorsed by the publisher.
